# Supplementary material for: Pre-exposure prophylaxis implementation during incarceration: Perspectives of formerly incarcerated men and women
Source: PLoS One. 2024 Dec 12;19(12):e0311289. doi: 10.1371/journal.pone.0311289 (PMC11637367; doi:10.1371/journal.pone.0311289)
Supplement: Attachment — Submitted filename: Response to Reviewers ver1.docx [file pone.0311289.s001.docx]

Dear Editor, Reviewers,

We thank you for your fair reviews and constructive suggestions. The authors appreciate the opportunity to submit a minor revision and hope that the manuscript is now suitable for publication. Below, we respond to each comment in **bold**. Line numbers refer to line numbers within the document titled “Manuscript with Track Changes.”

Sincerely,

On behalf of all authors,

Elizabeth Banyas

PONE-D-24-11121

Pre-exposure prophylaxis implementation during incarceration: perspectives of formerly incarcerated men and women

PLOS ONE

Dear Dr. White,

Thank you for submitting your manuscript to PLOS ONE. After careful consideration, we feel that it has merit but does not fully meet PLOS ONE’s publication criteria as it currently stands. Therefore, we invite you to submit a revised version of the manuscript that addresses the points raised during the review process.

Please submit your revised manuscript by Jul 24 2024 11:59PM. If you will need more time than this to complete your revisions, please reply to this message or contact the journal office at <a href="mailto:plosone@plos.org">plosone@plos.org. When you're ready to submit your revision, log on to https://www.editorialmanager.com/pone/ and select the 'Submissions Needing Revision' folder to locate your manuscript file.

Please include the following items when submitting your revised manuscript:

- A rebuttal letter that responds to each point raised by the academic editor and reviewer(s). You should upload this letter as a separate file labeled 'Response to Reviewers'.
- A marked-up copy of your manuscript that highlights changes made to the original version. You should upload this as a separate file labeled 'Revised Manuscript with Track Changes'.
- An unmarked version of your revised paper without tracked changes. You should upload this as a separate file labeled 'Manuscript'.

If you would like to make changes to your financial disclosure, please include your updated statement in your cover letter. Guidelines for resubmitting your figure files are available below the reviewer comments at the end of this letter.

If applicable, we recommend that you deposit your laboratory protocols in protocols.io to enhance the reproducibility of your results. Protocols.io assigns your protocol its own identifier (DOI) so that it can be cited independently in the future. For instructions see: <https://journals.plos.org/plosone/s/submission-guidelines#loc-laboratory-protocols>. Additionally, PLOS ONE offers an option for publishing peer-reviewed Lab Protocol articles, which describe protocols hosted on protocols.io. Read more information on sharing protocols at <https://plos.org/protocols?utm_medium=editorial-email&utm_source=authorletters&utm_campaign=protocols>.

We look forward to receiving your revised manuscript.

Kind regards,

Hamid Sharifi, Professor
Academic Editor
PLOS ONE

Journal Requirements:

When submitting your revision, we need you to address these additional requirements.

1. Please ensure that your manuscript meets PLOS ONE's style requirements, including those for file naming. The PLOS ONE style templates can be found at 
https://journals.plos.org/plosone/s/file?id=wjVg/PLOSOne_formatting_sample_main_body.pdf and 
https://journals.plos.org/plosone/s/file?id=ba62/PLOSOne_formatting_sample_title_authors_affiliations.pdf

2. We note that the grant information you provided in the ‘Funding Information’ and ‘Financial Disclosure’ sections do not match.

When you resubmit, please ensure that you provide the correct grant numbers for the awards you received for your study in the ‘Funding Information’ section.

3. Please review your reference list to ensure that it is complete and correct. If you have cited papers that have been retracted, please include the rationale for doing so in the manuscript text, or remove these references and replace them with relevant current references. Any changes to the reference list should be mentioned in the rebuttal letter that accompanies your revised manuscript. If you need to cite a retracted article, indicate the article’s retracted status in the References list and also include a citation and full reference for the retraction notice.

Additional Editor Comments:

Dear Authors,
Thanks so much for submitting this work to PLOS ONE. Both reviewers have recommended to accept manuscript. Howereve, before final decision, I have a few comments that should be apply.

1- The structure of the paragraphs should be improved. For example, the first two paragraphs of the introduction are only one sentece. Or the second last paragraph of the introduction should be impowered.

**The structure of the first paragraph within the introduction was adjusted [39]. We connected the first, single, standalone sentence within the following paragraph.**

**We also divided the second paragraph of the introduction into two paragraphs. The new second paragraphs focuses on HIV acquisition and risk prior to incarceration. The new third paragraph is focused on HIV transmission in correctional settings as previous.**

**The second to last paragraph within the introduction was improved in three ways. 1) The revised paragraph introduces the acronym ”PrEP” properly [58]. 2) The reference to Zambian prisons was removed as it does not relate to the scope of this paper [59]. 3) We explicitly state there is a ”gap between efficacy and availability” of PrEP. Citation numbers were adjusted accordingly within the manuscript and reference list.**

2- There is no need to add sutitles into the discussion.

**All subtitles within the discussion were removed.**

3- Please check and fix the references based on PLOS ONE format.

**All references now follow the PloS ONE format.**


[Note: HTML markup is below. Please do not edit.]

Reviewers' comments:

Reviewer's Responses to Questions

**Comments to the Author**

1. Is the manuscript technically sound, and do the data support the conclusions?

The manuscript must describe a technically sound piece of scientific research with data that supports the conclusions. Experiments must have been conducted rigorously, with appropriate controls, replication, and sample sizes. The conclusions must be drawn appropriately based on the data presented.

Reviewer #1: Partly

Reviewer #2: Yes

2. Has the statistical analysis been performed appropriately and rigorously?

Reviewer #1: Yes

Reviewer #2: Yes

3. Have the authors made all data underlying the findings in their manuscript fully available?

The [PLOS Data policy](http://www.plosone.org/static/policies.action#sharing) requires authors to make all data underlying the findings described in their manuscript fully available without restriction, with rare exception (please refer to the Data Availability Statement in the manuscript PDF file). The data should be provided as part of the manuscript or its supporting information, or deposited to a public repository. For example, in addition to summary statistics, the data points behind means, medians and variance measures should be available. If there are restrictions on publicly sharing data—e.g. participant privacy or use of data from a third party—those must be specified.

Reviewer #1: Yes

Reviewer #2: Yes

4. Is the manuscript presented in an intelligible fashion and written in standard English?

PLOS ONE does not copyedit accepted manuscripts, so the language in submitted articles must be clear, correct, and unambiguous. Any typographical or grammatical errors should be corrected at revision, so please note any specific errors here.

Reviewer #1: Yes

Reviewer #2: Yes

5. Review Comments to the Author

Please use the space provided to explain your answers to the questions above. You may also include additional comments for the author, including concerns about dual publication, research ethics, or publication ethics. (Please upload your review as an attachment if it exceeds 20,000 characters)

Reviewer #1: Thank you for your time and work in this very important area, and allowing me the opportunity to review your work.

I would advise that the author(s) add the transcript(s) of conversations as appendices to allow further context to the conversation. Despite the quotes being impactful and articulated well throughout, I feel that some of the power or context of the words of individuals may have been lost or missed in the bulk of the text.

**Thank you for your comment. To meet PloS ONE’s requirements for data availability, our submission includes a Data Availability Statement. The statement explains there are ethical restrictions on publishing a de-identified data set to the public. Thus, the authors believe the transcripts should not be included within an appendix to protect potentially sensitive information. However, researchers who meet the criteria to access confidential data are encouraged to contact the research ethics committee at UNC Chapel Hill to obtain access.**

This is an incredibly insightful written piece and it is very interesting to hear the views of those who are, or could be, directly affected - I appreciate that some of the conversations may have been triggering for individuals. Given the cultural differences outside of the U.S., it would be a great opportunity to build and expand on this piece of work in other countries and gain further insight into the understanding and provision of PrEP.

**Thank you for your comment.**

Reviewer #2: 1. Is the manuscript technically sound, and do the data support the conclusions?
The manuscript appears technically sound. The study is based on qualitative research and includes detailed descriptions of the methodology, data collection, and analysis processes. The conclusions drawn by the authors are supported by the data, which are well-documented and aligned with the study's objectives and findings. The discussion section logically connects the results to the broader context of HIV prevention among formerly incarcerated individuals, suggesting that the data adequately support the conclusions made.

**Thank you for your comment.**

2. Has the statistical analysis been performed appropriately and rigorously?
The manuscript employs qualitative research methods, and while it does not involve traditional statistical analysis, the qualitative data analysis is performed rigorously. Themes were derived from participant responses, and the authors followed a systematic approach to coding and interpreting the data. This method is appropriate given the research question and study design, ensuring the analysis is thorough and valid within the context of qualitative research.

**Thank you for your comment.**

3. Have the authors made all data underlying the findings in their manuscript fully available?
The manuscript includes a Data Availability Statement indicating that there are ethical restrictions on sharing a de-identified data set because the data contain potentially sensitive information. However, the contact information for the research ethics committee at UNC is provided for researchers who meet the criteria for access to confidential data. This meets the journal's requirements for data availability under conditions where full public sharing is not possible.

**Thank you for your comment.**

4. Is the manuscript presented in an intelligible fashion and written in standard English?
The manuscript is presented clearly and is written in standard English. The structure is logical, with a well-defined introduction, methodology, results, discussion, and conclusion. The language is professional and accessible, making the manuscript intelligible to a broad academic audience. The authors effectively communicate their findings and their significance to the field.

Overall, the manuscript is well-prepared and adheres to the standards expected for publication.

**Thank you for your comment.**

6. PLOS authors have the option to publish the peer review history of their article ([what does this mean?](https://journals.plos.org/plosone/s/editorial-and-peer-review-process#loc-peer-review-history)). If published, this will include your full peer review and any attached files.


If you choose “no”, your identity will remain anonymous but your review may still be made public.


**Do you want your identity to be public for this peer review?** For information about this choice, including consent withdrawal, please see our [Privacy Policy](https://www.plos.org/privacy-policy).

Reviewer #1: **Yes:** Geraint Jones

Reviewer #2: **Yes:** Ayalew Aklilu Haile
