# Supplementary material for: Pre-exposure prophylaxis implementation during incarceration: Perspectives of formerly incarcerated men and women
Source: PLoS One. 2024 Dec 12;19(12):e0311289. doi: 10.1371/journal.pone.0311289 (PMC11637367; doi:10.1371/journal.pone.0311289)
Supplement: Attachment — Submitted filename: Response to Reviewers 9_12_24.docx [file pone.0311289.s002.docx]

THE UNIVERSITY OF NORTH CAROLINA AT CHAPEL HILL

**T** 919.966.2537 | **F** 919.966.6714

Bioinformatics Building | Floor 2 | Campus Box 7030

130 Mason Farm Road

Chapel Hill, NC 27599-3368

globalhealth.unc.edu

September 12 2024

Dear Editors and Reviewers,

We thank you for your fair reviews and constructive suggestions. The authors appreciate the opportunity to submit a minor revision and hope that the manuscript is now suitable for publication. Below, we respond to each comment in **bold**. Line numbers refer to line numbers within the document titled “Manuscript with Track Changes.”

*Editor Comment #1 (from first revision): The structure of the paragraphs should be improved. For example, the first two paragraphs of the introduction are only one sentence. Or the second last paragraph of the introduction should be impowered.*

**The structure of the first paragraph within the introduction was adjusted [39]. We connected the first, single, standalone sentence within the following paragraph.**

**We also divided the second paragraph of the introduction into two paragraphs. The new second paragraph focuses on HIV acquisition and risk prior to incarceration. The new third paragraph is focused on HIV transmission in correctional settings as previous.**

**The second to last paragraph within the introduction was improved in three ways. The revised paragraph introduces the acronym ‘‘PrEP”.) The reference to Zambian prisons was removed as it does not relate to the scope of this paper . We explicitly state there is a gap between efficacy and availability of PrEP. Citation numbers were adjusted accordingly within the manuscript and reference list.**

*Editor Comment #2 (from first revision): There is no need to add subtitles into the discussion.*

**All subtitles within the discussion are removed.**

*Editor Comment #3 (from first revision): Please check and fix the references based on PLOS ONE format.*

**All references now follow the PloS ONE format.**

*Editor Comment #1 (from second revision): Using this new released guideline, please revise some words like prisoners or incarcerated people.*

**We appreciate you for providing the UNAIDS terminology guidelines. We went through the manuscript and updated any terms no longer in use to preferred terms.**

*Editor Comment #2 (from second revision): The previous submitted file did not include the previous comments and also the responses of the authors. I need a cover letter including the previous comments and also the responses of the author how they did apply the comments.*

**We hope that this document containing comments and responses meets the standards of PLoS One.**

*Reviewer Comment #1: Thank you for your time and work in this very important area, and allowing me the opportunity to review your work. I would advise that the author(s) add the transcript(s) of conversations as appendices to allow further context to the conversation. Despite the quotes being impactful and articulated well throughout, I feel that some of the power or context of the words of individuals may have been lost or missed in the bulk of the text.*

**Thank you for your comment. To meet PloS ONE’s requirements for data availability, our submission includes a Data Availability Statement. The statement explains there are ethical restrictions on publishing a de-identified data set to the public. Thus, the authors believe the transcripts should not be included within an appendix to protect potentially sensitive information. However, researchers who meet the criteria to access confidential data are encouraged to contact the research ethics committee at UNC Chapel Hill to obtain access.**

Sincerely yours,

Sincerely,

Becky L. White MD MPH,

Associate Professor of Medicine,

Division of Infectious Diseases

University of North Carolina at Chapel Hill School of medicine

[bls@med.unc.edu](mailto:bls@med.unc.edu), 9199662536, 9199666714
